# Functional and Metabolic Characterization of Microglia Culture in a Defined Medium

**DOI:** 10.3389/fncel.2020.00022

**Published:** 2020-02-07

**Authors:** Alejandro Montilla, Alazne Zabala, Carlos Matute, María Domercq

**Affiliations:** ^1^Department of Neurosciences, University of the Basque Country, Leioa, Spain; ^2^Achucarro Basque Center for Neuroscience-UPV/EHU, Leioa, Spain; ^3^Instituto de Salud Carlos III, Centro de Investigación Biomédica en Red de Enfermedades Neurodegenerativas (CIBERNED), Leioa, Spain

**Keywords:** microglia, culture, ramification, phagocytosis, metabolic reprogramming, purinergic receptors, motility

## Abstract

Microglia are the endogenous immune cells of the brain and act as sensor of infection and pathologic injury to the brain, leading to a rapid plastic process of activation that culminates in the endocytosis and phagocytosis of damaged tissue. Microglia cells are the most plastic cells in the brain. Microglia isolation from their environment as well as culturing them in the presence of serum alter their function and lead to a rapid loss of their signature gene expression. Previous studies have identified pivotal factors allowing microglia culture in the absence of serum. Here, we have further characterized the function, expression of markers, metabolic status and response to pro and anti-inflammatory stimulus of microglia isolated by magnetic-activated cell sorting and cultured in a chemically defined medium. We have compared this new method with previous traditional protocols of culturing microglia that use high concentrations of serum.

## Introduction

Microglial cells are the resident macrophages of the central nervous system (CNS). They arise from early myeloid progenitors in the embryonic yolk sac that migrate and colonize CNS (Ginhoux et al., 2010; Kierdorf et al., 2013). These progenitors expand, proliferate and differentiate into parenchymal microglia and their lineage is preserved through continuous self-renewal even in pathological conditions (Ajami et al., 2011). The other CNS non-parenchymal macrophages, including perivascular, meningeal and choroid plexus macrophages, share with parenchymal microglia the prenatal origin, the lack of replacement by circulating blood cells, and major parts of the transcriptional profile (Goldmann et al., 2016).

Microglia are characterized by a continuous surveillance of their environment, in order to maintain tissue integrity and homeostasis (Davalos et al., 2005; Nimmerjahn et al., 2005). Moreover, they play a pivotal role in a wide and heterogeneous range of physiological responses, actively interacting with almost all CNS cell types. These functions vary during the different stages of development, and they transcend the expected immunity-specific activities such as phagocytosis of myelin debris or apoptotic cells. Microglia is involved during development in circuit refinement through synaptic pruning (Schafer et al., 2012) and in myelogenesis (Wlodarczyk et al., 2017). In the adult, microglia is involved in maintaining the homeostasis of the baseline neurogenic cascade through the elimination of neural precursor cells (NPCs; Sierra et al., 2010).

Microglial cells are widely studied in pathological conditions as well, as its activation is thought to contribute to neuroinflammation, a crucial hallmark of neurodegenerative disorders such as Alzheimer’s disease, Parkinson’s disease or multiple sclerosis (MS). These diverse and sometimes contradictory functions in which microglia/macrophages are involved can be explained on the basis of a broad spectrum of activation states, depending on the nature of the activating stimulus (Xue et al., 2014). These different activation states are associated to a metabolic reprogramming. Similar to macrophages (Kelly and O’Neill, 2015; Stienstra et al., 2017), microglia exposed to pro-inflammatory stimulus shift their metabolism from oxidative phosphorylation (OXPHOS) to aerobic glycolysis (Hu et al., 2019; Nair et al., 2019), in an event similar to the Warburg effect suffered by cancer cells (Andrejeva and Rathmell, 2017). This metabolic reprogramming is essential to activate cellular defense mechanisms and to manage various microenvironments in inflamed tissue (Kelly and O’Neill, 2015; Van den Bossche et al., 2017; Holland et al., 2018). In contrast, macrophages exposed to anti-inflammatory stimulus are primarily characterized by oxidative phosphorylation and increased fatty acid oxidation for ATP synthesis (Stienstra et al., 2017; Van den Bossche et al., 2017), a fact that it has not been corroborated in microglia cells.

The endeavor to understand microglial functions and specifications has led to the development of both *in vivo* and *in vitro* models. The former include microglial deficient mice, whereas the latter comprise immortalized cell lines like BV-2 or primary microglial cultures. Among the primary cultures, microglia isolated from mixed glial cell cultures are among the most common ones. Nevertheless, almost all *in vitro* models present a drawback: they are performed in the presence of serum to support cell survival. Microglia in these cultures are highly proliferative and display an amoeboid morphology, typical of the injured brain, and differ from highly ramified surveillant microglia. In order to take over that limitation, Bohlen et al. (2017) developed a serum-free medium in which primary microglia could survive at higher rates and develop a ramified morphology that resembles that acquired by these cells in the normal adult brain (Bohlen et al., 2017). To achieve so, they identified three factors, TGF-β2, IL-34, and cholesterol (TIC factors), essential to their survival. Here, we have gone a step forward to characterize morphologically and functionally these primary cells isolated by Magnetic-activated cell sorting (MACS) from postnatal rat brain and cultured in the presence of a defined medium containing TIC factors, and to determine whether these cells can recapitulate the microglial metabolic reprogramming described before in response to stimulation with pro-inflammatory factors. In comparison to microglia derived from mixed glial cell culture with serum-supplemented medium, microglia growth in the chemically defined medium showed a downregulation of almost all the activation markers analyzed. Moreover, microglial phagocytic activity is lowered even though their motility is enhanced. Importantly, we observed that these cells can be activated and reprogram their metabolism.

## Methods

### Animals

All experiments were performed according to the procedures approved by the Ethics Committee of the University of the Basque Country (UPV/EHU). Animals were handled in accordance with the European Communities Council Directive. All possible efforts were made to minimize animal suffering and the number of animals used.

### Serum-Exposed Microglia Isolation and Culture

Primary mixed glial cultures were prepared from the cerebral cortex of neonatal rats (P0-P2). After 10–15 days in culture, microglia were isolated by mechanical shaking (400 rpm, 1 h) and purified by plating on non-coated bacterial grade Petri dishes (Sterilin; Thermo Fisher) as previously described (Domercq et al., 2007). Microglial cells obtained with this procedure were cultured in Dulbecco’s Modified Eagle Medium (DMEM; Gibco) supplemented with 10% Fetal Bovine Serum (FBS; Gibco). We have selected this protocol for culturing microglia, the most common one, despite the existence of other methods using lower concentration of serum and mild trypsinization (Saura et al., 2003).

Microglial cells were stimulated with pro-inflammatory or anti-inflammatory factors as previously described with minor modifications (Zabala et al., 2018). Briefly, cells were treated during 24 h with pro-inflammatory factors LPS (10 ng/ml) plus IFN-γ (20 ng/ml) or with the anti-inflammatory cytokines interleukins IL-13 (50 ng/ml) and IL-4 (20 ng/ml).

### MACS Isolated Microglia Culture

Microglial cells were isolated by MACS from P10-P12 rats, following a protocol similar to that stablished in the Neural Tissue Dissociation Kit P datasheet (Miltenyi Biotec). Briefly, the whole brain (without cerebellum) was carefully dissected and meninges were removed. Subsequently, the brain tissue was dissociated both enzymatically and mechanically using the indicated enzymatic mixes and a dounce homogenizer, respectively. Myelin was removed in order to increase the yield using a Percoll gradient protocol, and finally CD11b^+^ cells were sorted by MACS using species-specific microbeads (Miltenyi Biotec). Approximately 2–3 million cells were obtained from a single brain.

These cells were subsequently resuspended and cultured in a chemically defined medium containing TGF-β2, cholesterol and macrophage colony-stimulating factor (M-CSF; 100 ng/ml, Peprotech) (TIC factors), as previously described with minor modifications (Bohlen et al., 2017). In our case, IL-34 was replaced with M-CSF that acts via the same signaling pathway (CSF1R). Microglial cells were cultured with a variable density depending on the experiment; normally, in order to increase the outcome of functional cells, the density of the culture was high (30,000–60,000 cells per well). Medium was refreshed every day until the cells were used/analyzed. Cell purity was analyzed using antibody markers for astrocytes (GFAP; 1:100, Millipore), neurons (NeuN; 1:500, Millipore) and oligodendrocytes (Olig2; 1:200, Millipore).

Cells obtained through the MACS protocol were stimulated with proinflammatroy factors LPS (10 ng/ml) plus IFN-γ (20 ng/ml) or with the anti-inflammatory cytokines interleukins IL-13 and IL-4 at low concentration (low; IL-13, 50 ng/ml and IL-4, 20 ng/ml) or high concentration (high; IL-13, 100 ng/ml and IL-4, 50 ng/ml).

### Immunofluorescence Analysis

In order to characterize MACS-isolated primary microglia, several immunocytochemistry (ICC) assays were performed on them as well as on traditional microglia cultures in the presence of serum. Specific ICC were performed in order to assay the expression of activation markers, purinergic receptors, transcription factors and markers of autophagy lysosomes and lipid bodies. Cells were fixed in 4% p-formaldehyde (PFA) in PBS and processed for ICC as previously described (Domercq et al., 1999). Primary antibodies were used as follows to: iNOS (1:500, BD Bioscience), MRC1 (1:1000, Abcam), CD68 (1:100, Bio-Rad), Iba1 (1:500, Wako), IRF5 (1:100, Millipore), P2 × 7R (1:100, Alomone), P2 × 4R (1:400, Alomone), P2Y12 (1:200, Anaspec) and LC3B (1:2000, Novus Biologicals). Lipid bodies (LBs) were stained using BODIPY 493/503 (Invitrogen). As secondary antibodies, we used goat anti-rabbit Alexa Fluor 488 (1:250, Invitrogen), goat anti-rabbit Alexa Fluor 594 (1:250, Invitrogen), goat anti-mouse Alexa Fluor 594 (1:250, Invitrogen) and goat anti-rat Alexa Fluor 488 (1:250, Invitrogen). Images were acquired using a laser scanning confocal Olympus Fluoview FW500 microscopy or a Leica TCS STED CW SP8 super-resolution microscope, using the same settings for all samples within one experimental group.

All the image analysis was performed with the ImageJ software (NIH). Morphology analysis of microglia was performed with ImageJ software as described before (Fontainhas et al., 2011). The area of the cell was determined on the basis of Iba1 immunostaining using NIH ImageJ. The number of processes arising from soma was quantified manually. Both parameters were analyzed in 30–50 cells in three independent experiments. Mean fluorescence intensity (fluorescence intensity/cell area, as defined in ImageJ) of purinergic receptors, IRF5, iNOS, MNR, and Bodipy labeling was calculated in individual cells, defined on the basis of Iba1 immunostaining (data was obtained from 20–30 cells per coverslip from 3–5 different experiments performed in duplicate). Regarding IRF5, the mean intensity (IRF5 fluorescence/ROI area) was calculated in defined ROIs in cytoplasm and nucleus and the results were expressed as the ratio (mean fluorescence cytoplasm/mean fluorescence nucleus). CD68^+^ and LC3^+^ puncta per cell were quantified at individual cells in 20–30 cells from three independent cultures.

### Phagocytosis Assays

To evaluate phagocytosis, microglia were incubated with fluorescent microbeads (FluoSpheres^TM^ carboxylate-modified microspheres, 2 μm, red fluorescent (580/605); Invitrogen) for 1 h at 37°C, rinsed, and fixed with 4% PFA. Cells were stained using antibodies to Iba1 (1:500; Wako) and Hoechst 33258. Fluorescent microbeads were quantified using ImageJ on microglial cells outlined with the Iba1 immunostaining as the defining parameter for the ROIs. An intracellular section of the cell was selected on the basis of Iba1 staining to assure intracellular location of the microbeads. Identical acquisition parameters were used for image capture of individual experiments. Results were expressed as the percentage of beads phagocytosed versus total beads (10 fields/experiment, *n* = 3 independent experiments) and the percentage of phagocytic cells (≥1 bead, at least 50 cells were analyzed in each experiment, *n* = 3 independent experiments).

Furthermore, phagocytic capacities were also assessed by measuring the internalization and degradation of myelin. With that in mind, myelin was isolated from adult rat spinal cord with sucrose gradient centrifugations (Norton and Poduslo, 1973), and labeled with Alexa 488-NHS dye (A2000 Life Technologies) for 1 h at RT in PBS. Subsequent dialysis helped removing dye excess, and the resulting dyed myelin was stored until its addition to the microglia cultures in a final concentration of 5 μg/mL (1:200 dilution). To evaluate myelin endocytosis, the cells were rinsed and fixed after 1 h of incubation at 37°C. On the other hand, in order to evaluate myelin degradation by the lysosomes, the excess of myelin was removed and cells were subsequently chased after 24 h. Microglia were then stained using antibodies to Iba1 and Hoechst 33258, and myelin was quantified at both conditions on Iba1^+^ cells using ImageJ (data was obtained from at least 50 cells/experiment from three independent experiments).

### Metabolism Analysis

Real time measurements of OCR and ECAR were performed using a Seahorse XFe96 Extracellular Flux Analyzer (Agilent). Microglia cells were seeded as a monolayer in a 96-well microplate and the analysis was performed following the manufacturer instructions. Before the assay, cells were washed and equilibrated in the XF Assay modified DMEM medium for 30 min at 37°C. The real levels of oxygen consumption rate were determined in response to the sequential addition of oligomycin (2 mM), FCCP (1 mM) and rotenone/antimycin A (0.5 mM). Basal mitochondrial respiration was calculated as the last measurement before addition of oligomycin – non-mitochondrial respiration (minimum rate measurement after Rot/AntA). Spare capacity was calculated by subtracting basal respiration from the maximum rate measurement after addition of FCCP. Estimated ATP production designated the last measurement before addition of oligomycin – the minimum rate after oligomycin. All these parameters were obtained using the Agilent Report Generator.

### Microglial Dynamics Analysis

For these purposes, cells were cultured in 35 mm Glass Bottom Dishes (MatTek Corporation). Following 2 or 3 days of incubation at 37°C, the dishes were transferred to the Nikon Biostation IM-Q microscope, and live images of cells were captured with the time lapse imaging system to study microglial dynamics. The settings for the recording were as follows: 3 h with an acquisition of a frame per minute. The tracking of the different cells acquired in those time-lapses were obtained using the Kinovea software (*n* = 20 cells from three independent experiments). The accumulated distance traveled by the individual cells was quantified by measuring the euclidean distances covered at every frame; subsequently, the mean speed of the cells was calculated dividing these distances by the time.

## Results

### Microglial Morphology and Phenotype Changes in a Chemically Defined Medium

Using CD11b-microbeads for the isolation protocol, microglia cells were isolated with a high purity from postnatal rat brains. The vast majority of these cells (purity > 99%) were positive for the phagocyte marker Iba1, whereas practically no labeling of astrocytes (<1%), oligodendrocytes or neurons was observed in our cultures.

**FIGURE 1:**
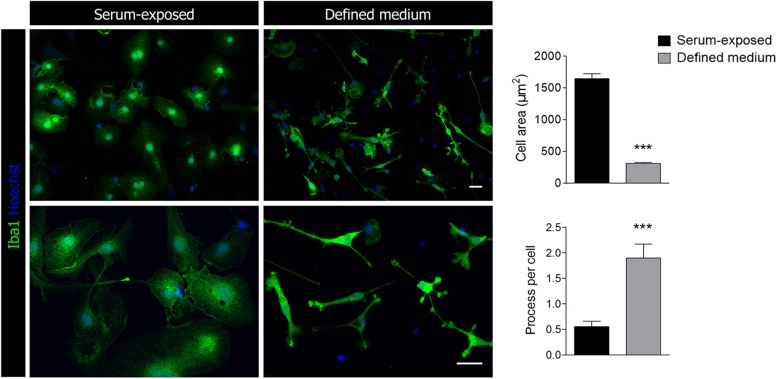
Representative images of Iba1^+^ microglia cultured in the presence of serum or in a chemically defined medium. Scale bar = 25 μm. Histograms represent the morphological characterization of microglia in defined medium and serum-exposed microglia. Data shows mean ±s.e.m. of 20–30 cells from three different experiments. *** *p* < 0.001, Student’s *t*-test.

Morphological analysis based on Iba1 immunostaining showed a significant increase in the number of processes and a decrease in the cell body area in microglia cultured in defined medium (Figure 1). Thus, microglia acquired a highly ramified morphology, with multiple processes branching off the soma, resembling the typical microglia morphology presented in physiological conditions in the brain. In contrast, those cells cultured with serum presented an amoeboid-like phenotype (Figure 1).

**FIGURE 2:**
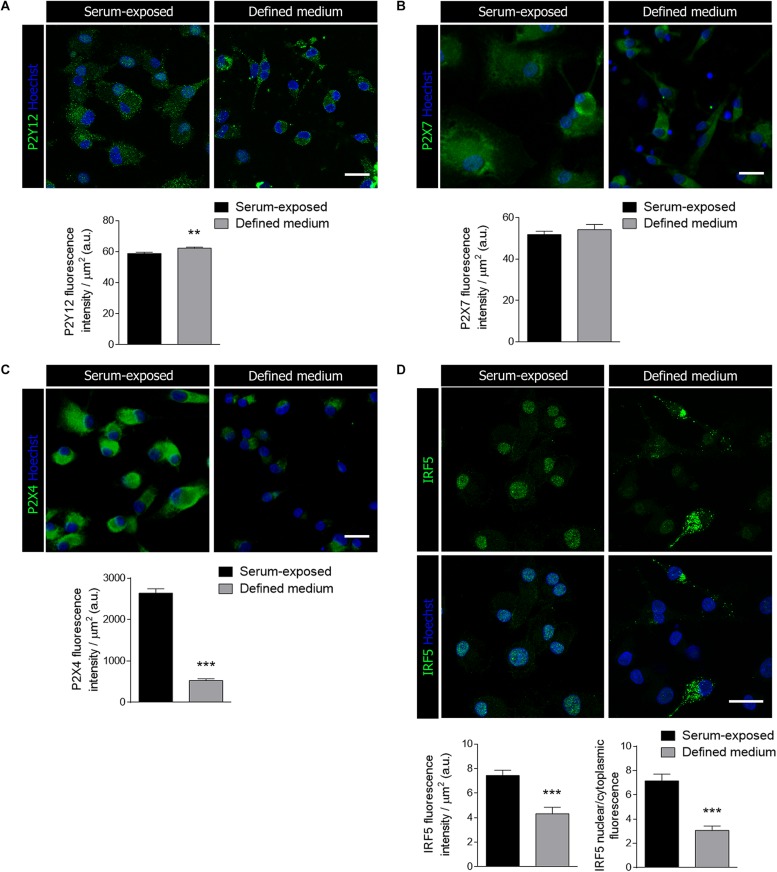
Microglial purinergic signaling was modified when cultured in the defined medium. **(A–C)** Immunostaining of the purinergic receptors P2Y12 (A), P2 × 7 **(B)** and P2 × 4 **(C)**, all of them associated to microglial activation and function, in serum-exposed microglia and microglia cultured in defined medium. Scale bars = 20 μm. Histograms represent the mean ± s.e.m of the P2 receptors fluorescence intensity per cell. **(D)** Immunostaining of the interferon regulatory factor 5 (IRF5), a transcription factor that controls immune response and the expression of P2 × 4, in serum-exposed microglia and microglia in defined medium. Scale bars = 20 μm. Histograms show the quantification of the immunoreactivity and the ratio of IRF5 nuclear translocation in serum-exposed microglia and microglia cultured in defined medium. Data shows mean ± s.e.m. of 20–30 cells per coverslip from 3–5 different experiments performed in duplicate. ** *p* < 0.01, *** *p* < 0.001, Student’s *t*-test.

Since purinergic receptors, particularly P2Y12, P2 × 4 and P2 × 7, are pivotal in microglial activation and function (Clark et al., 2010; Beaino et al., 2017; Zabala et al., 2018), we checked whether the presence of serum influences purinergic receptor immunoreactivity. Microglia activation is associated to specific gene signatures, such as P2Y12 downregulation and P2 × 4 overexpression (Domercq et al., 2010). No change was detected in P2 × 7R expression between both culture conditions (Figure 2B). In contrast, we detected a significant increase in P2Y12R immunoreactivity and a drastic downregulation of P2 × 4R immunoreactivity in microglia cultured in defined medium (Figures 2A,C). Since P2 × 4R expression is controlled by the transcription factor interferon regulatory factor-5 (IRF5) (Masuda et al., 2014), a key regulator of the inflammatory reaction, we analyzed IRF5 immunoreactivity in both populations. Total IRF5 immunoreactivity was reduced in microglia cultured in defined medium. Moreover, microglia cultured in defined medium showed a reduction in nuclear IRF5 immunoreactivity, as analyzed by the nucleus/cytoplasm labeling ratio (Figure 2D), suggesting a decreased level of IRF5-targeted genes transcription.

### Phagocytosis and Surveillance

**FIGURE 3:**
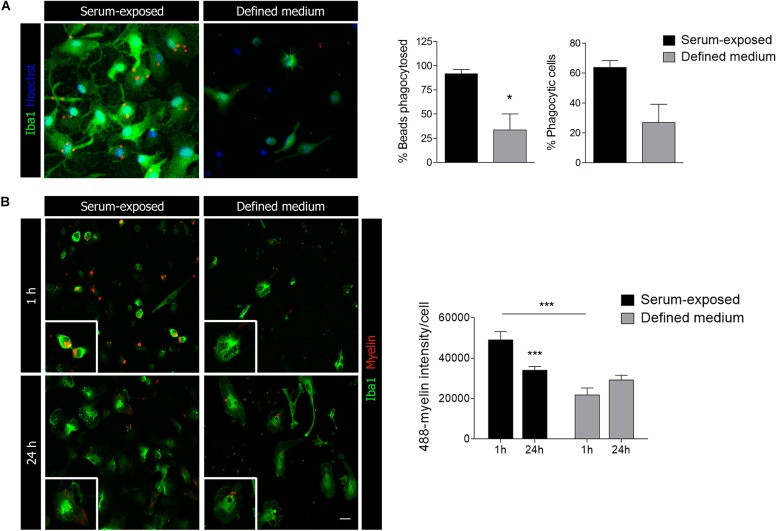
Microglial phagocytic capacity is reduced in microglia cultured in defined medium. **(A)** Phagocytosis of fluorescent microbeads (1 h) in microglia cultured in defined medium and serum-exposed microglia. Scale bar = 20 μm. Histograms show the percentage of beads taken up by the cells (vs. total beads) and the percentage of phagocytic cells. **(B)** Alexa-488-labeled myelin endocytosis at 1 h (top) and 24 h (bottom) in serum-exposed microglia and microglia in defined medium. Scale bar = 20 μm. Histogram shows myelin endocytosis (1 h) and degradation (24 h) in both conditions. Notice that microglia cultured in defined medium do not degrade myelin. Data shows mean ± s.e.m. of at least 50 cells in each experiment, *n* = 3 independent experiments performed in duplicate. * *p* < 0.05, *** *p* < 0.001, Student’s *t*-test.

In light of the previously mentioned results, we explored whether primary microglia basic functionalities were altered as well in the absence of serum. Firstly, phagocytosis was assessed treating the cells with specific fluorescent microbeads for an hour pre-fixation. We observed a significant decrease in the percentage of microbeads phagocytosed per cell as well in the number of phagocytic cells in microglia cultured in defined medium (Figure 3A). However, and in contrast to results obtained previously (Bohlen et al., 2017), microglia cultured in defined medium were still able to phagocyte microbeads (not opsonized) although less efficiently. To further explore phagocytosis, we isolated myelin from adult rat whole brain using sucrose gradient (Norton and Poduslo, 1973), labeled with Alexa 488 and added to microglial cells. We quantified myelin internalization and delivery to lysosomes for degradation. As with microbeads, we observed a significant reduction on myelin endocytosis at 1 h in microglia cultured in defined medium (Figure 3B). Moreover, myelin in microglia cultured in defined medium was not reduced at 24 h (Figure 3B), indicating a failure or a less efficient degradation capacity of these microglia.

**FIGURE 4:**
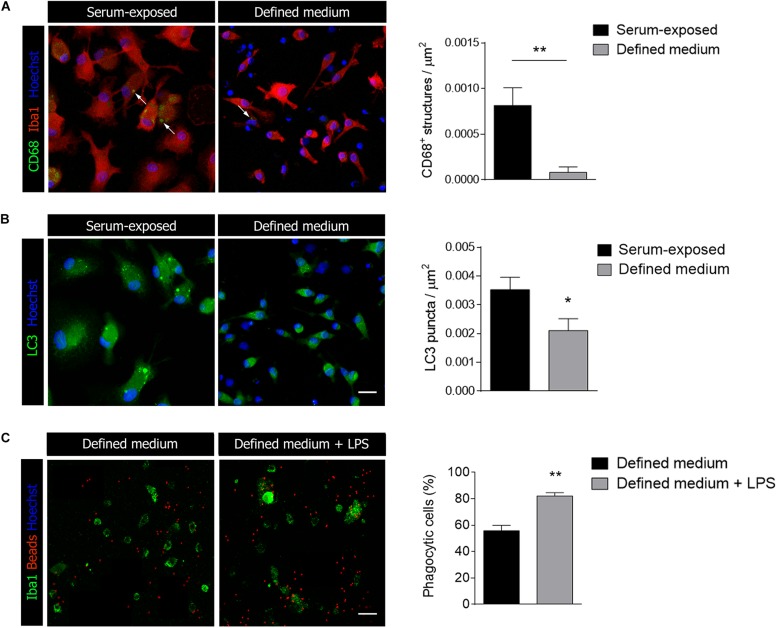
Microglial activation markers are reduced in defined medium. **(A,B)** Immunostaining of CD68 **(A)**, a lysosomal marker related to activation and LC3 **(B)**, a marker of autophagy, in serum-exposed microglia and in microglia culture in defined medium. Data was obtained from at least 20 cells in each experiment, *n* = 3 independent experiments performed in duplicate. **(C)** Phagocytosis of fluorescent microbeads (24 h) in defined medium without and with exposure to LPS. Scale bar = 20 μm. Histogram shows the percentage of phagocytic cells in both cases. Data was obtained from at least 50 cells in each experiment, *n* = 3 independent experiments. Data shows mean ± s.e.m. * *p* < 0.05, ** *p* < 0.01, Student’s *t*-test.

In relation with phagocytosis, we also tested markers of autophagy and lysosomes. CD68 is a protein found on lysosomal membrane that is associated to the cell’s phagocytic activity. We found CD68 immunoreactivity to be severely reduced in microglia cultured in defined medium (Figure 4A). We further analyzed microtubule-associated protein 1A/1B–light chain 3 (LC3) immunoreactivity. In myeloid cells, LC3-associated phagocytosis (LAP), a non-canonical type of autophagy, couples the processing of engulfed particles, including pathogens, immune complexes, and dying cells, to regulation of macrophage immune responses (Cunha et al., 2018). LC3 immunostaining was also reduced in microglia cultured in defined medium (Figure 4B). Altogether, data indicated that microglia cultured in the defined medium have reduced phagocytosis.

Microglia exposed to pro-inflammatory stimuli such as LPS increases phagocytosis (Rangaraju et al., 2018) although surveillant microglia constantly move their processes to patrol the environment and phagocytose apoptotic cells and synapses during development (Sierra et al., 2010; Paolicelli et al., 2011). In order to check whether stimulation with pro-inflammatory factors increases phagocytosis in defined medium microglia, we treated cells with LPS at a concentration that does not affect significantly cell survival (100 ng/ml; 24 h) and immediately tested microglia phagocytosis. We observed a significant increase in the percentage of phagocytic cells when exposed to fluorescent microbeads (Figure 4C).

**FIGURE 5:**
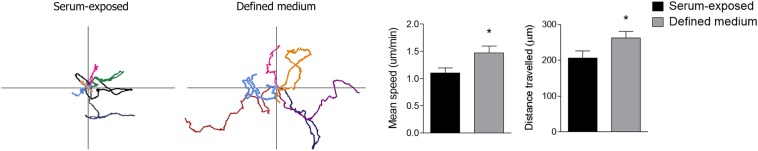
Microglial surveillant capacity is increased when cultured in defined medium. Representative trajectories of single cells (left) are represented as differentially colored lines. The point of intersection of the axes constitute the initial position of the cells. The histogram (right) represent the mean speed of their movement. Both parameters were acquired using the Kinovea software. The time-lapse video (3 h, one frame per minute) was obtained using a Nikon Biostation IM-Q microscope. Data shows mean ± s.e.m. from at least 20 cells/experiment, *n* = 3 independent experiments. * *p* < 0.05, Student’s *t*-test.

In the normal physiological condition, microglia scan constantly brain parenchyma by constant process movement. Live imaging of the microglia cells demonstrated that microglia cultured in defined medium have higher motility. The mean speed of the cells was enhanced and cells moved longer distances (Figure 5 and Supplementary Videos). These data support the idea that the phenotype of microglia cultured with defined medium actually resembles more the one present in the brain in physiological conditions.

### Polarization and Metabolic Reprogramming

**FIGURE 6:**
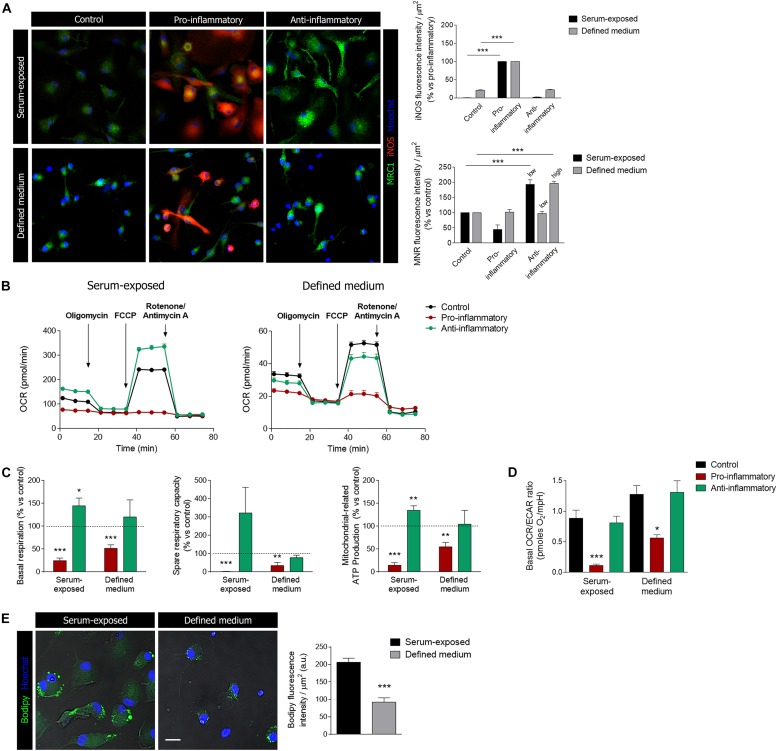
Microglia activation and metabolic reprogramming in microglia cultured in defined medium. **(A)** Staining for iNOS (red) and mannose receptor (MRC1, green) in differentially activated microglia (see methods) in serum-exposed microglia and in microglia cultured in defined medium. Notice that anti-inflammatory factors were used at two concentration, low and high (see details in section “Methods”), in microglia cultured in defined medium whereas the low concentration of stimulus was only efficient in serum-exposed microglia. Scale bar = 50 μm. Data shows mean ± s.e.m. of at least 20 cells/experiment, *n* = 3 independent experiments. *** *p* < 0.001, ANOVA and Bonferroni *post hoc* analysis. **(B)** One representative experiment showing the metabolic profiles in microglia in defined medium and serum-exposed microglia after incubation with pro-inflammatory or anti-inflammatory factors for 24 h. **(C)** Metabolic parameters related to mitochondrial respiration in both microglia exposed to serum and microglia cultures in defined medium, after stimulation with pro-inflammatory or anti-inflammatory factors for 24 h. Histograms represent the normalization of these parameters with respect to the control cells (mean + s.e.m.; *n* = 5–8 independent experiments). * *p* < 0.05, ** *p* < 0.01, *** *p* < 0.001, Student’s *t*-test. **(D)** OCR/ECAR ratio obtained from the basal levels of each parameter. Histogram represent mean + s.e.m. of *n* = 5–8 independent experiments. For each experiment, 6 to 8 replicates were used. * *p* < 0.05, *** *p* < 0.001, ANOVA and Bonferroni *post hoc* analysis. **(E)** Lipid accumulation in lipid bodies (LBs) represented by the expression of the fluorescent probe Bodipy, in serum-exposed microglia and microglia cultured in defined medium. *** *p* < 0.001, Student’s *t*-test. Error bars in all the histograms represent standard error of the mean (s.e.m.).

Microglia display an enormous plasticity in their responses to injury, ranging from responses that contribute to damage to responses essential for regeneration and inflammation resolution (Fumagalli et al., 2018). Much of this work has been performed *in vitro* using protocols to activate cells toward a pro-inflammatory phenotype and toward an anti-inflammatory and regenerative phenotype. Microglia cells were primed pro-inflammatory and anti-inflammatory factors (see details in section “Methods”) and analyzed by immunocytochemistry using pro-inflammatory (iNOS) and anti-inflammatory (mannose receptor; MRC1) markers. Stimulation with pro-inflammatory factors, LPS (10 ng/ml) + IFNγ (20 ng/ml), induced a significant increase in iNOS immunoreactivity in both culture models (Figure 6A). In contrast, anti-inflammatory cytokines IL-13 and IL-4 induced an increase in MRC1 immunoreactivity in serum-exposed microglia at low concentrations (50 and 20 ng/ml, respectively; Figure 6A) but not in microglia cultured in defined medium (Figure 6A). Higher concentrations of anti-inflammatory cytokines IL-13 and IL-4 (100 and 50 ng/ml, respectively) were needed to observe an increase in MRC1 immunoreactivity in microglia cultured in defined medium (Figure 6A).

Microglia stimulation with anti or pro-inflammatory factors is accompanied by metabolic reprogramming (Kelly and O’Neill, 2015). Thus, microglia treated with pro-inflammatory stimulus would mainly rely on the glycolytic pathway to generate the energy in the form of ATP. We assessed the energetic profiles of microglia treated with anti- and pro-inflammatory factors in both culture conditions using the Mito Stress Kit in a Seahorse XFe96 Analyzer (Agilent), in order to know if in both culture conditions reprogramming is maintained.

Serum-exposed microglia treated with classical pro-inflammatory stimuli (LPS and IFN-γ) showed a flat profile regarding oxidative phosphorylation (OXPHOS), and all the parameters (basal OCR, ATP production-associated OCR and spare respiratory capacity) related to this molecular process were practically abolished (Figures 6B,C). This suggest a metabolism based mostly on the glycolytic process, in order to rapidly obtain energy. Similarly, microglia cultured in defined medium showed a significant reduction, but not abolishment, in the same parameters of mitochondrial respiration (Figures 6B,C). On the other hand, serum-exposed microglia stimulated with anti-inflammatory factors showed a significant increase in basal mitochondrial respiration and in the spare capacity (Figures 6B,C). In contrast, mitochondrial metabolism did not significantly change in microglia cultured in defined medium after exposure to anti-inflammatory factors. We further checked glycolysis in both culture models. The ratio OCR/ECAR was significantly decreased after exposure to pro-inflammatory factors in both culture models (Figure 6D), thus indicating that microglia shift to a glycolitic phenotype.

Finally, we used the fluorescent probe Bodipy to characterize the lipid accumulation in lipid bodies (LBs) in both microglia culture models. LBs, also referred to lipid droplets, are inducible cytoplasmic organelles containing neutral lipids (triacylglycerol, diacylglycerol or steryl esters) with roles in regulation of lipid metabolism, cell signaling and activation, membrane trafficking and control of the synthesis and secretion of inflammatory mediators Pro-inflammatory stimuli such as LPS induce an accumulation of LBs and an enhancement of their size in microglia (Khatchadourian et al., 2012). Similar to other markers, we observed a significant reduction in LBs in microglia cultured in defined medium versus serum-exposed microglia (Figure 6E).

## Discussion

Here we have characterized primary microglia isolated by magnetic sorting and cultured in a chemically defined medium with previously identified factors that promote microglial survival (Bohlen et al., 2017). This condition is known to provoke a ramified morphology that mirrors that acquired by these cells *in vivo* in the normal adult brain. Besides, we identify the obtained phenotype as a more representative example of resting microglia observed in physiological conditions, ranging from a downregulation of activation markers to a reduction of different functionalities typically associated to the activated state of microglia, including phagocytosis. Importantly, the capacity of the cells to polarize to pro-inflammatory or anti-inflammatory profiles when exposed to common agents, as well as the metabolic reprogramming that cells suffer during this process remains mainly unaltered.

Serum-free culture conditions are essential to reproduce the physiological condition of the extracellular fluid of the CNS, which is equal to the cerebrospinal fluid. Normal cerebrospinal fluid (CSF) contains extremely low levels of proteins and bioactive factors. In this study, we corroborated that the chemically defined medium more closely reproduce the *in vivo* extracellular medium than the medium containing high percentage of serum and lead to a morphological and functional microglia state resembling homeostatic microglia *in vivo*. Moreover, the increased dynamics of microglia cultured in defined medium also mimics the ones observed in physiological conditions. Microglia functions are highly regulated *in vivo*. Microglial activation is accompanied by transcriptional reorganization of different signaling receptors, in particular purinergic receptors. ATP is one of the most important and ancient danger signal to alert and recruit immune cells to the site of tissue damage following injury and to orchestrate host immunity and inflammation. In this sense, ATP is one of the main regulators of microglial functions. Low ATP concentrations almost exclusively activate chemotaxis, through P2Y12 receptor activation, in order to recruit cells at the site of injury or inflammation (Haynes et al., 2006). When the ATP concentration increases, additional effector functions, such as phagocytosis and cytokine secretion, are also triggered (Domercq et al., 2018). The switch on microglial ATP responses are sometimes linked to an acute remodeling of purinoceptor expression. Specifically, when microglia is activated by pro-inflammatory factors or after pathological stimulus, they upregulate P2 × 4 whereas P2Y12 receptors expression is decreased (Koizumi et al., 2013; Domercq et al., 2018). Whereas minor changes were observed in P2 × 7 expression between both microglia models, P2Y12 levels were higher and P2 × 4 was dramatically downregulated in microglia cultured in the absence of serum in the defined medium, probably as a result of IRF5 transcription factor downregulation. Importantly, this receptor is quickly activated after injury in different pathological conditions, defining the P2 × 4R^+^ reactive microglia (Masuda et al., 2014). These results suggest that microglia cultured in defined medium have purinergic sensome characteristics of homeostatic microglia *in vivo* (Hickman et al., 2013).

One of the functions of microglia is to phagocytose cellular debris, myelin and pathogens. Microglia cultured in defined medium showed lower phagocytic capacity of fluorescent beads or myelin than microglia cultured in the presence of serum, as previously described (Bohlen et al., 2017). Moreover, we detected that myelin degradation at lysosomes is totally impaired in microglia cultured in defined medium. Accordingly, the number of CD68^+^ lysosomes or LC3 puncta is also severely reduced in this model. It is well known that serum increases microglia phagocytosis *in vitro* (Bohlen et al., 2017) as well as *in vivo*. Indeed, lesions accomplished with blood brain barrier alterations showed higher phagocytic activity in microglia (Schilling et al., 2005; Greenhalgh and David, 2014). Thus, the lower phagocytic activity of microglia in this optimized culture conditions resembles homeostatic microglia *in vivo*. Although this model could have some limitations to study phagocytosis function *in vitro*, it could be used on the other hand to study and identify factors and signaling pathways that potentiate phagocytosis with possible therapeutic potential.

Immunometabolism is a fast growing field of immunology. Macrophages and microglia undergo profound metabolic reprogramming in response to environmental cues, such as hypoxia, nutrient alterations, and in response to danger signals and cytokines. In our study, cells cultured in defined medium also decrease the OCR/ECAR ratio after exposure to proinflammatory activators such as LPS and IFNγ, demonstrating that microglia undergo a metabolic reprogramming under these conditions. In contrast, microglia cultured in defined medium did not show a clear increase in basal OXPHOS nor in the spare respiratory capacity of the OXPHOS after exposure to anti-inflammatory cytokines (IL-4 and IL-13). Altogether, data suggest that stimulation with anti-inflammatory factors did not induce any metabolic change in microglia cultured in defined medium. These difference with regards to serum-exposed microglia may suggest that microglia in defined medium are slightly shifted toward an anti-inflammatory phenotype (Cherry et al., 2014). Further metabolic characterization of MACS isolated microglia and cultured in this conditions would shed light on the metabolic signature of homeostatic microglia *in vivo*.

## Conclusion

Magnetic-activated cell sorting isolated microglia and cultured in a chemically defined medium without serum is a useful but limited model to study microglia physiology. Receptor expression is more representative of microglia in physiological conditions and thus, its characterization would help to define more accurately the signaling pathways that orchestrate microglia function and activation. This model would also serve to study metabolic reprogramming secondary to microglia stimulation with pro-inflammatory factors; however, it is somehow limited for phagocytic studies. In particular, the machinery to degrade myelin seems to be impaired in microglia in this culture model.

## Data Availability Statement

The datasets generated for this study are available on request to the corresponding author.

## Ethics Statement

The animal study was reviewed and approved by the University of the Basque Country (Spain) Animal Ethics Committee.

## Author Contributions

MD contributed to the conception and design of the study. AM and AZ performed the experiments. AM and MD wrote the sections of the manuscript. All authors contributed to the manuscript revision, read, and approved the submitted version.

## Conflict of Interest

The authors declare that the research was conducted in the absence of any commercial or financial relationships that could be construed as a potential conflict of interest.

## Supplementary Material

VIDEO S1 Cell motility of microglia cultured without serum. Phase images of P12 microglia at 3 div. Each frame is 1 min apart and movies are played at 7.2 frames per second.

VIDEO S2 Cell motility of microglia cultured with serum. Phase images of P0 microglia. Each frame is 1 min apart and movies are played at 7.2 frames per second.
